# The role of relational support in the longitudinal links between adolescent sexual harassment victimization and psychological health

**DOI:** 10.1017/S0954579420000565

**Published:** 2021-10

**Authors:** Therése Skoog, Sabina Kapetanovic

**Affiliations:** 1 Department of Psychology, University of Gothenburg, Goteborg, Sweden; 2 University West, Trollhattan, Sweden

**Keywords:** adolescence, emotional problems, relationship support, sexual harassment, well-being

## Abstract

The links between sexual harassment victimization and aspects of psychopathology are well-established in adolescent research, but whether sexual harassment victimization undermines positive aspects of psychological health and the moderating role of relational support in the link between sexual harassment victimization and psychological ill-health remains unknown. Using a cross-lagged model, we examined (a) the bidirectional and longitudinal links between sexual harassment victimization and adolescent psychological health (emotional problems and well-being) and (b) the moderating role of relational support from parents, teachers, and peers (best friends and classmates) in the link between sexual harassment victimization and adolescent psychological health. We used two waves of self-reported data (separated by 1 year) from 676 Swedish adolescents (50% female; mean age = 13.85 years at the point of first data collection). Controlling for the effects of gender and subjective socioeconomic status, the cross-lagged model revealed that sexual harassment predicted emotional problems positively and well-being negatively. Moreover, well-being predicted sexual harassment negatively. Relational support from classmates moderated the link in the direction from sexual harassment victimization to emotional problems. Relational support did not moderate the link to well-being. The findings provide new and important insights into the role of sexual harassment victimization in adolescent psychological adjustment and potential approaches to intervention.

Adolescent sexual harassment, defined broadly as “unwanted sexual attention” (McMaster, Connolly, Pepler, & Craig, 2002, p. 92), includes sexual comments, grabbing, and touching. It becomes increasingly common in early adolescence after the onset of puberty (Petersen & Hyde, 2009). Peer sexual harassment is clearly distressing (Hill & Kearl, 2011) and many researchers have studied and identified links between sexual harassment victimization and psychological health problems, including depressive symptoms and self-harm (Dahlqvist, Landstedt, Young, & Gådin, 2016; Marshall, Faaborg-Andersen, Tilton-Weaver, & Stattin, 2013; Petersen & Hyde, 2013; Skoog, Bayram Özdemir, & Stattin, 2016). At the same time, whether sexual harassment undermines adolescent psychological well-being remains unknown. Moreover, developmental theory (e.g., Sameroff, 2010) suggests that adolescent psychosocial development affects and is affected by mutual and co-occurring developmental processes. Thus, in theory, being sexually harassed may have negative consequences for adolescent psychological health, but poor adolescent psychological health may also be of importance for the occurrence of sexual harassment. However, little research attention has been paid to the mutual and bidirectional influences of sexual harassment victimization on aspects of psychological illness and health. Furthermore, scant has been paid to identifying moderating factors that may influence the links between sexual harassment victimization and adolescents’ poor psychological health.

## Sexual harassment and links to adolescents’ psychological health

Sexual harassment is a common form of peer victimization in adolescence. Half or more of adolescents in Europe and the USA report having been the target of sexual harassment (Petersen & Hyde, 2009; Vega-Gea, Ortega-Ruiz, & Sánchez, 2016; Young, Grey, & Boyd, 2009). In a nationally representative sample of adolescents in seventh to twelfth grade in the USA, 48% reported having been the victims of sexual harassment during the course of one school year, with verbal harassment being the most common form (Hill & Kearl, 2011). Although several studies have found that girls are more exposed to sexual harassment than boys (Hill & Kearl, 2011; Ormerod, Collinsworth, & Perry, 2008), findings about gender differences in sexual harassment victimization are inconsistent. A large-scale Finnish survey of 180,000 14- to 18-year-olds reported that 40% of all boys and 55% of all girls had experienced some form of sexual harassment at some time (Kaltiala-Heino, Frojd, & Marttunen, 2016). Others have found that boys are more exposed (Li, Frieze, & Tang, 2010). In Spain, 63% of boys and 53% of girls (mean age 16.8 years) reported having been the targets of visual/verbal sexual harassment over the previous 3 months (Vega-Gea et al., 2016). However, the difference in exposure between genders is generally small or even non-existent (Ashbaughm & Cornell, 2008). Apparently, sexual harassment is part of an adolescent's social contexts regardless of their gender.

The literature has identified a clear link between sexual harassment victimization and the development of psychopathology among adolescents. In early adolescence, the particular importance of interactions with peers increases (Bornstein, Jager, & Steinberg, 2013). Heightened social concern means that adolescents become highly attuned to how peers think of them and treat them (Somerville, 2013). Consequently, adolescents are at particularly high risk of psychological harm when victimized by peers. Accordingly, longitudinal studies in different contexts converge to show that sexual harassment victimization by peers predicts psychological health problems among adolescents, including depressive symptoms and self-harm (Bendixen, Daveronis, & Kennair, 2018; Chiodo, Wolfe, Crooks, Hughes, & Jaffe, 2009; Dahlqvist et al., 2016; Hatchel, Espelage, & Huang, 2018; Petersen & Hyde, 2013; Rinehart, Espelage, & Bub, 2017; Skoog et al., 2016). Because of the focus in the literature on aspects of psychological problems or illness, whether sexual harassment undermines adolescent psychological well-being remains unknown. However, the link between sexual harassment victimization and poor psychological health is robust and holds even after accounting for other risk factors, including low socioeconomic status (SES) and ethnic background (Bendixen et al., 2018). Concern regarding its impacts is fueled by the fact that having emotional problems in adolescence increases the risk of psychological health problems being sustained into adulthood (Johnson, Dupuis, Piche, Clayborne, & Colman, 2018). Given the vast number of victimized adolescents and the well-established negative psychological consequences, sexual harassment among adolescent peers is a serious public health threat.

Although developmental theory (e.g., Sameroff, 2010) states that adolescent psychosocial development affects and is affected by different co-occurring and reciprocal developmental processes, little research attention has been paid to examining whether adolescents’ (poor) psychological health has an effect on their risk of being sexually harassed. The focus has been on the unidirectional link from sexual harassment to poor psychological health, particularly depressive symptoms. This has contributed to a limited understanding of the interrelationship between sexual harassment and poor psychological health. Scholars suggest that depressive symptoms may elicit negative reactions from adolescents’ peers because such symptoms signal vulnerability (Schacter & Juvonen, 2017). Accordingly, less than a handful of longitudinal studies have demonstrated that depressive symptoms (Dahlqvist et al., 2016) and self-harm (Marshall et al., 2013) predict the future risk of being sexually harassed, although not among sexual minority youth (Hatchel et al., 2018). Most studies in the field use cross-sectional data, but those studies do not allow for conclusions about the temporal sequence of sexual harassment victimization and psychological health.

## The protective role of relational support

Although sexual harassment is clearly distressing, there is individual variability in adolescents’ responses to it. This suggests that there may be moderating factors that alter the risk of developing psychological health problems when subjected to sexual harassment. Theoretically, moderating factors could also alter the risk of being sexually harassed when experiencing poor psychological health. In the last three decades there has been a growing recognition of the concept of resilience in adolescent development (Masten & Barnes, 2018; Masten, Best, & Garmezy, 1990). Resilience is “the process of, capacity for, or outcome of successful adaptation despite challenging or threatening circumstances” (Masten et al., 1990, p. 426). Resilience requires the presence of protective factors—known as assets and resources—when confronted with environmental stressors or individual vulnerabilities. Whereas assets reside within the individual, resources are part of the environment. Relational support is fundamental and a central part of individuals’ resources. The resilience literature posits that “The capacity of an individual to adapt to challenges depends on their connections to other people” (Masten & Barnes, 2018, p. 2). The oft-cited buffering effect hypothesis makes the same prediction (Cohen & Wills, 1985). Consequently, relational support might have the potential to attenuate the negative psychological consequences of being sexually harassed or decrease the risk of being sexually harassed for psychologically vulnerable adolescents.

Parents, teachers, and peers are the main sources of relational support in adolescents’ developmental ecologies. According to developmental theories (Bronfenbrenner, 1995; Sameroff, 2010), adolescent development cannot be separated from its social context, where parents, peers, and teachers constitute the microsystems in which adolescents grow and develop. In other words, adolescent development is a product of dynamic interactions with the systems within which adolescents are included. For example, parents are considered a proximal part of a child's context, playing a key role in the development of their children (Darling & Steinberg, 1993; Gariépy, Honkaniemi, & Quesnel-Vallée, 2016). As suggested by Bowlby (1978), parents and children form emotional bonds with each other, which constitute the base of children's development and internal working models of themselves and the social world they are in. These bonds are moderately to highly stable over the course of childhood and adolescence (Cortés-García, Wichstrøm, Viddal, & Senra, 2019), which indicates that the support and emotional closeness that parents provide is beneficial even during the transition to adolescence. At this stage in life, peers take up more space in adolescents’ lives (Bornstein et al., 2013). Depending on the quality of their relationships with peers, adolescents are likely to experience positive or negative developmental outcomes (e.g., Burk & Laursen, 2005; Hiatt, Laursen, Mooney, & Rubin, 2015). In addition, adolescents spend a large amount of time in schools, where they extend their personal ecologies to include classmates and teachers (Hendrickx, Mainhard, Boor-Klip, Cillessen, & Brekelmans, 2016). Teachers facilitate the development of social cognitions and generally promote healthy interactions among students (Pianta, Hamre, & Stuhlman, 2003). Thus, parents, peers, and teachers have important roles in adolescent development.

The literature on adolescent sexual harassment has supported the idea of relational support being central in adolescent development by revealing that adolescents with stronger relational support from peers, parents, and people in school are less likely to be sexually harassed (Doty, Gower, Rudi, McMorris, & Borowsky, 2017; Espelage et al., 2019; Gruber & Fineran, 2016; Kaltiala-Heino et al., 2016). For instance, in a large-scale study, Doty et al. (2017) showed that being sexually harassed was negatively associated with the quality of connections with both parents and teachers. Similarly, Gruber and Fineran (2016) reported that sexually harassed 15-year-olds had less support from teachers than their non-sexually harassed peers. Evidently, relational support plays a protective role in sexual harassment victimization in adolescence. What is still unknown, however, is if relational support alters the link between sexual harassment victimization and poor psychological health.

We propose that it does. Specifically, we propose that the quality of adolescents’ relational support moderates the link in the direction from peer sexual harassment victimization to poor psychological health. We argue that when adolescent victims of peer sexual harassment are embedded in relationship systems with social support, they will develop less severe emotional problems and experience higher well-being than if they lack relational support. The study reported in this paper focused on relational support from parents, teachers, best friends, and classmates. Although parents are arguably children's most important source of relational support (Gariépy et al., 2016), sexual harassment among adolescents most commonly occurs outside of the home. School is a common arena for sexual harassment (Young et al., 2009), where teachers and peers (classmates) are. Relational support from teachers and peers at school might play the most important protective roles in the link between sexual harassment victimization and psychological symptoms. They could be a resource in terms of providing immediate help to deal with the stress caused by harassment, and other peers could make adolescents feel less alienated or alone when sexually victimized in their peer group.

The literature on the protective role of relational support in the link between general peer victimization and psychological health outcomes (Gariépy et al., 2016; Santini, Koyanagi, Tyrovolas, Mason, & Haro, 2015) supports our proposition. Several studies have found that relational support from significant others (e.g., family and peers) moderates the link between bullying victimization and psychological symptoms (Elgar et al., 2014; Ostrov & Kamper, 2015; Sapouna & Wolke, 2013; Thompson & Leadbeater, 2013). In line with such findings, studies have found that adolescents who are victimized by peers—online or offline—have higher well-being if they have high-quality friendships, particularly in terms of perceived support from friends or good relationships with classmates (Cuadros & Berger, 2016; Davidson & Demaray, 2007; Frison, Subrahmanyam, & Eggermont, 2016). Others have found that relational support from peers, but not parents, moderates the link between bullying victimization and emotional problems (Holt & Espelage, 2007). Moreover, relational support from school staff has been found to moderate the link between bullying victimization and emotional problems (Duong & Bradshaw, 2014). There are thus both theoretical and empirical reasons for believing that relational support moderates the link between sexual harassment victimization and poor psychological health. Nevertheless, this proposition has never been directly tested in studies. Understanding the possible protective role of relational support is fundamental to designing effective interventions aimed at reducing the adverse impact of sexual harassment victimization on adolescents’ psychological health.

## The present study

In summary, fundamental questions remain unanswered concerning the links between peer sexual harassment victimization and adolescents’ psychological health. Firstly, the literature takes a predominantly psychopathological perspective. Links between sexual harassment victimization and aspects of psychological health have been neglected. Secondly, developmental theory (e.g., Sameroff, 2010) states that adolescent psychosocial development affects and is affected by different co-occurring and reciprocal developmental processes. However, there is little evidence concerning the predictive value of psychological problems and health for sexual harassment victimization. Thirdly, theory (Masten & Barnes, 2018; Masten et al., 1990) and related empirical findings (Cohen & Wills, 1985; Cuadros & Berger, 2016; Davidson & Demaray, 2007; Duong & Bradshaw, 2014; Eccles & Roeser, 2011; Elgar et al., 2014) emphasize relational support as a key resource in adolescent development, particularly under stressful and challenging circumstances such as being victimized by peers at school. Relationships with parents, teachers, and peers are the most important sources of relational support for adolescents. However, we are unaware of studies that have examined relational support from parents, teachers, and peers as moderators of the robust link between sexual harassment victimization and poor psychological health among adolescents. In light of this, the current study was designed with two main aims.

The first aim was to investigate the short-term longitudinal and bidirectional links between sexual harassment victimization and two main aspects of psychological health, specifically emotional problems and subjective well-being (i.e., life-satisfaction and purpose and meaning in life), in early adolescence. Based on the literature review, we hypothesized that sexual harassment victimization is positively associated with emotional problems and negatively associated with subjective well-being. Given that most previous studies do not allow for conclusions about the temporal sequence of sexual harassment victimization and poor psychological health, we had no hypotheses regarding the direction of these effects. By using longitudinal data and studying bidirectional links, the current study had the potential to shed new light on the interrelationship between sexual harassment victimization and aspects of both emotional problems and well-being. Despite the major role of digital environments in adolescents’ social worlds, school is still one of the most common arenas for sexual harassment among adolescents, and peers are the most common offenders in this context (Espelage, Hong, Rinehart, & Doshi, 2016; Young et al., 2009). Therefore, the current study focused on peer sexual harassment victimization that occurs in the school context. The development period in focus was early adolescence because the prevalence of sexual harassment increases markedly with puberty (Petersen & Hyde, 2009; Skoog & Bayram Özdemir, 2016a, 2016b). In fact, sexual harassment can be seen as part of, or rather a consequence of, the bio-psychosocial contexts of early adolescence due to the developmental tasks of this period such as the formation of sexual identity, sexual orientation, and emerging romantic relationships.

The second aim was to explore the moderating roles of relational support from parents, teachers, and peers (best friends and classmates) in the mutual and short-term longitudinal links between sexual harassment victimization and poor psychological health. We hypothesized that the quality of adolescents’ relational support moderates the link in the direction from peer sexual harassment victimization to emotional problems and low subjective well-being. We controlled statistically for the part played by gender and subjective SES in all the analyses.

Data from Sweden was used in the present study. Sweden is ranked as one of the countries in the world with the greatest gender equity (Gaye, Klugman, & Kovacevic, 2010), but reports show that it is also the part of the European Union with the highest prevalence of sexual harassment (Fundamental Rights Agency, 2014). Moreover, Swedish youth show a high prevalence of negative psychological symptoms, and symptom levels are increasing more in Sweden than in other European countries (Bremberg, 2015). Consequently, Sweden provides an interesting and important context for the study of adolescent sexual harassment and links to poor psychological health among adolescents (Gådin & Stein, 2019).

## Methods

### Participants

Data were taken from the Swedish research program Longitudinal Research on Development In Adolescence (LoRDIA; Kapetanovic, Skoog, Bohlin, & Gerdner, 2019). LoRDIA studies transitions in adolescence by collecting information about adolescents’ health, school functioning, relations with family, teachers, and peers, and the development of risk behaviors such as substance use and delinquency. The program is designed to follow adolescents in four medium-sized municipalities in southern Sweden for 4–5 years, from 12/13 to 18 years of age. Data collection started in 2013 with two cohorts, students in sixth and seventh grade. Out of a total of 2,150 invited students in Wave 1 and Wave 2, 18% opted out, resulting in a total study population of 1,886 students. The last data collection took place in 2018, when the older cohort of adolescents were in their second year of senior high school.

The measures used in LoRDIA varied somewhat between waves. This means that some data were only collected once or twice. The sample for the current study is based on two waves of self-reported data, separated by 1 year, from one cohort of adolescents who by the third wave of the study (here referred to as Time 1 (T1)) were in eighth grade. In total, 676 adolescents with a baseline mean age of 13.85 years (*SD* = 0.41) were included. The sample was evenly divided by gender, with 340 boys (50.3%) and 336 girls (49.7%). Most were of Swedish ethnicity (79.6%) and lived with both parents (72.5%). LoRDIA has no objective measure of SES, but most adolescents in the analytical sample (71.2%) reported having as much money as their classmates (10.3% reported that their family had more, while 17.5% that they had less).

### Measures

#### Sexual harassment

The items related to sexual harassment were placed in a questionnaire section that focused on peer behaviors at school. This measure assessed experiences of sexual harassment during the most recent school semester using two items: “Has anyone commented on your looks or your body in a sexual way that you didn't like?” and “Has anyone fondled or touched your body in a sexual way that you didn't like?” The items were rated from 1 (= *No, never*) to 3 (= *Yes, many times*). The Spearman–Brown coefficient is the recommended reliability statistic for two-item scales (Eisinga, Grotenhuis, & Pelzer, 2013). The Spearman–Brown coefficients for Time 1 (T1) and Time 2 (T2) were .47 and .66, respectively. The inter-item correlations for T1 and T2 were .30 and .49, respectively.

#### Emotional problems

This measure is one of five subscales in the Swedish self-report version of the Strength and Difficulties Questionnaire (SDQ-S) (Lundh, Wångby-Lundh, & Bjärehed, 2008). The subscale assessed emotional problems using five items: “I worry a lot,” “I get a lot of headaches, stomach aches or sickness,” “I am often unhappy, down-hearted or tearful,” “I am nervous in new situations, I easily lose confidence,” and “I have many fears, I am easily scared.” The ratings ranged from 1 (= *Not true*) to 3 (= *Completely true*), with the following internal consistencies: T1, α = .77; T2, α = .60.

#### Well-being

This measure assessed adolescents’ life satisfaction and purpose and meaning in life (Berlin, Modin, Gustafsson, Hjern, & Bergström, 2012). It consisted of two questions: “In general, how satisfied are you with your life at the moment?” with ratings ranging from 1 (= *Very happy*) to 4 (= *Very unhappy*) and “I think that my life has purpose and meaning” with ratings ranging from 1 (= *Completely agree*) to 4 (= *Completely disagree*). The responses were later reversed so that higher values indicated higher well-being. The T1 and T2 Spearman–Brown coefficients for the scale were .76 and .74, respectively. The inter-item correlations for T1 and T2 were .62 and .59, respectively.

To provide a quantitative sense of the effects of relational support on adolescent psychosocial outcomes, the following measures were dichotomized and used as grouping variables in multi-group analyses. A median split was used to dichotomize scores into high and low scores. The most moderating measures were collected at T1.

#### Support from mother

This measure (Biesecker, 2007) assessed adolescent perceptions of emotional closeness and relational support from their mothers when adolescents were approximately 12 years of age (mean age  = 12.53 years; *SD* = 0.32) during the first wave of LoRDIA (i.e., approximately 1.5 years before T1). Five items were used: “I feel comfortable sharing my private thoughts and feelings with my mother,” “When I am angry or sad my mother can help me feel better,” “I know that my mother is there when I need her,” “My mother encourages me to pursue my dreams,” and “I feel that I can try new things because I know my mother supports me.” Answer options were 1 (= *Yes*), 2 (= *Sometimes*) and 3 (= *No, never*). The measure was internally consistent (α = .79). The structure of the questions was similar to that used in the Inventory of Parent and Peer Attachment (IPPA) (Armsden & Greenberg, 1987), which has been found to have satisfactory and high stability over the course of early to mid-adolescence (β = .73) (Cortés-García et al., 2019).

#### Support from teachers

This measure was adapted from the work of Kerr and Stattin (2000) and assessed adolescents’ perceptions of relational support from teachers using three questions: “Do the teachers at your school care about the students?” “Are the teachers at your school fair to the students?,” and “Do the teachers at your school like the students?” Ratings ranged from 1 (= *Yes, all or almost all teachers*) to 4 (= *No, hardly anyone*) with internal consistency (α = .87).

#### Support from classmates

This measure was developed for the LoRDIA project and assessed adolescents’ perceptions of relational support from classmates using four statements, all beginning with “In my class… .” The different statement ends were: “… we help each other out,” “… we like doing things together,” “… we are nice to each other,” and “… nobody is excluded.” Ratings ranged from 1 (*=* *Yes, completely true*) to 4 (*=* *Not true at all*) with internal consistency (α = .82). The coding was later reversed. It should be noted that in Sweden and at the ages of the participants, students spend most, but not all, of their day at school with the same group of students (i.e., their class).

#### Support from best friend

Perceptions of the quality of the relationship with and support from a best friend was measured using a modified version of the Friendship Quality Questionnaire (Kendrick, Jutengren, & Stattin, 2012; Parker & Asher, 1993). Having a supportive and trusting relationship was assessed using six items, such as “My best friend would not share my secrets with other people.” The ratings ranged from 1 (*=* *Not true*) to 3 (*=* *Yes, true*) with internal consistency (α = .65).

#### Demographics

Adolescent gender, family structure and SES at T1 were included in analyses as covariates and predictors of T2 adolescent psychosocial outcomes. Adolescent gender and family structure were measured via categorical variables. Adolescent gender was entered as “1” for female or “2” for male. Family structure was entered as “1” for living with both mother and father, “2” for alternating between mother and father, and “3” for adolescents living only with mother or father. Adolescent SES (Quon & McGrath, 2014) was calculated by averaging two items: “Do you perceive having less, as much, or more money than your classmates?” and “Does your family have less, as much, or more money than other families in your neighborhood?” The Spearman–Brown coefficient was .52 and the inter-item correlation was .35.

### Procedure

In 2013, contact was established with all primary schools in the participating municipalities and with parents of the students. Both students and their parents were informed about the study, confidentiality, and voluntary participation. Both parents and students had the opportunity to not give their consent for participation. The students replied annually to questionnaires, which were administered in classrooms by the research team. The research program and data collection details were approved by the Regional Research Review Board in Gothenburg, Sweden.

### Data analysis

**Figure 1.**
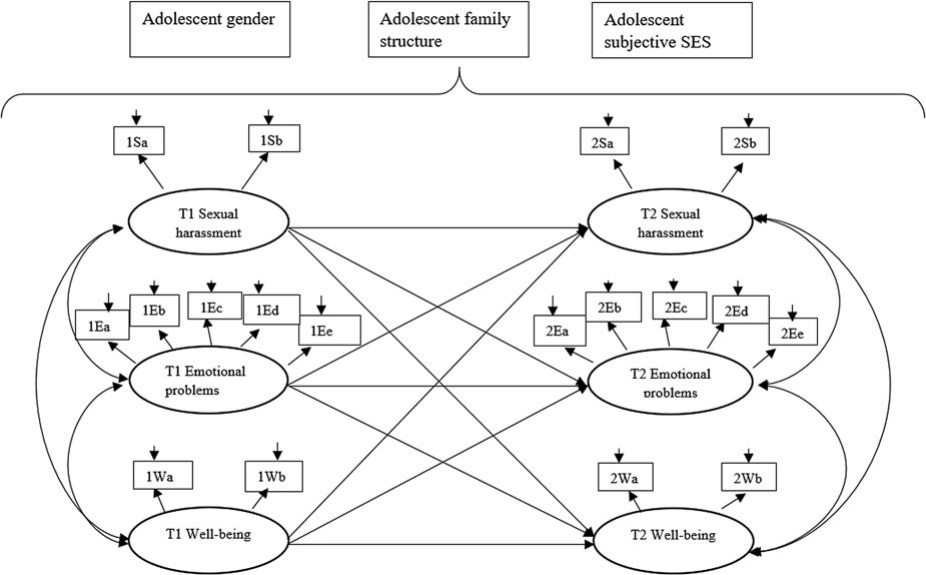
Conceptual model of links between latent factors of sexual harassment victimization, emotional problems and well-being, controlling for the effects of adolescent gender, family structure and subjective socioeconomic status (SES).

We calculated bivariate correlations between the study measures and conducted a series of independent *t* tests to test whether sexual harassment, emotional problems, and well-being differed between adolescent boys and girls. We then implemented structural equation modelling using AMOS 23.0 in four steps. Firstly, we conducted a series of confirmatory factor analyses (CFAs) to confirm the internal structure of the scales. After the CFAs were completed, for each measure, we compared models with unconstrained and constrained factor loadings over time in order to test measurement invariance. The relative fit of the constrained model was evaluated based on change in CFI (ΔCFI). The change in each model was <.01, which indicated an equivalent fit between the models (Van de Schoot, Lugtig, & Hox, 2012). We could therefore pursue further analyses. We fitted an integrated measurement model with six latent constructs: T1 and T2 sexual harassment, T1 and T2 emotional problems, and T1 and T2 well-being. Before proceeding with the analyses, we screened the data for missing values. Little's missing completely at random (MCAR) was significant, however the normed chi-square (χ^2^/*df*) was low (564.229/453 = 1.24), implying a small violation of the MCAR assumption. Therefore, the full information maximum likelihood procedure was used to handle missing data, making it possible to produce unbiased parameter estimates and bias-corrected confidence intervals (Byrne, 2010). We evaluated goodness-of-fit using χ^2^ (*p* > .05), the Tucker–Lewis index (TLI > .95), the comparative fit index (CFI > .90), and the root mean square error of approximation (RMSEA < .08). χ^2^ is known to be sensitive to sample size and too often produces statistically significant values (Hair, Black, Babin, Anderson, & Tatham, 2010), which is why the additional goodness-of-fit measures were employed. The baseline measurement model (Model 1) provided a good fit to the data (see Table 1). Then, we fitted a cross-lagged structural model (Model 2) to the latent variables to examine the relations between sexual harassment, emotional problems, and well-being. In the following step (Model 3), we added adolescent gender, family structure, and subjective economic status as correlates of T1 sexual harassment, T1 emotional problems, and T1 well-being, and predictors of T2 sexual harassment, T2 emotional problems, and T2 well-being to the cross-lagged structural model (see Figure 1 for the conceptual model). To obtain the most parsimonious model, we constrained the factor loadings in the constructs to be the same across time points (Model 4). Finally, we conducted a series of multi-group analyses to test whether the links between the latent constructs were moderated by support from the mother, support from teachers, support from classmates, and support from a best friend. A constrained model, where effects are equivalent across groups, and an unconstrained model, with freely varying effects, were compared using χ^2^-difference tests. The significantly better fit of the unconstrained model (as indicated by significant Δχ^2^) would indicate a moderation effect (Byrne, 2010).

**Table 1.** Goodness-of-fit indices

	*χ*^2^	*df*	*p*	TLI	CFI	RMSEA
Model 1	237.472	110	.000	.963	.973	.041
Model 2	218.978	110	.000	.967	.976	.038
Model 3	241.793	134	.000	.963	.977	.035
Model 4	272.970	152	.000	.965	.975	.034

## Results

Means, standard deviations, and *t* test values for the main measures are shown in Table 2. The *t* tests revealed that girls reported significantly higher sexual harassment at T1 and T2 than boys. Girls also reported higher levels of emotional problems at T1 and T2 than boys. Boys reported significantly higher well-being at T1 and T2 than girls. The bivariate correlations are provided in Table 3. The correlation analyses revealed several significant positive and negative bivariate correlations. For the sake of space, we will only describe correlations related to the outcome variables. Adolescent subjective SES was positively correlated with T1 and T2 well-being, and negatively correlated with T1 and T2 emotional problems. Furthermore, T1 support from the mother and T1 support from teachers were positively correlated with T1 and T2 well-being, and negatively correlated with T1 and T2 sexual harassment and T1 emotional problems. Support from teachers at T1 was also negatively correlated with T2 emotional problems. Support from classmates at T1 was also positively correlated with T1 and T2 well-being, and negatively correlated with T1 sexual harassment and T1 and T2 emotional problems. A T1 supportive peer relationship was positively correlated with T1 well-being. Finally, sexual harassment was positively correlated with emotional problems and negatively correlated with well-being at both points in time. In addition, emotional problems and well-being were negatively correlated at both points in time.

**Table 2.** Means, standard deviations, and *t* test values for boys’ and girls’ sexual harassment victimization, emotional problems, and well-being

	Girls (*n* = 336)		Boys (*n* = 340)		
	*M*	*SD*	*M*	*SD*	*t* (*df*)
T1 Sexual harassment	1.20	0.36	1.09	.25	4.45 (600.43) ***
T1 Emotional problems	1.80	0.51	1.41	.41	10.58 (633.16) ***
T1 Well-being	3.23	0.68	3.49	.59	−5.20 (655.78) ***
T2 Sexual harassment	1.23	0.41	1.10	.35	4.06 (559.97) ***
T2 Emotional problems	1.86	0.50	1.46	.43	10.30 (577.09) ***
T2 Well-being	3.16	0.70	3.44	.63	−5.07 (593) ***

*Note*: *** *p* < .001.

**Table 3.** Zero-order correlations among main variables

	Adolescent gender	Family structure	Subjective SES	T1 Support from mother	T1 Support from classmates	T1 Support from teachers	T1 Support from best friend	T1 Sexual harassment	T1 Emotional problems	T1 Well-being	T2 Sexual harassment	T2 Emotional problems
Family structure	.04											
Subjective SES	−.02	−.15**										
T1 Support from mother	−.06	−.04	.15**									
T1 Support from classmates	.10*	−.03	.05	.07								
T1 Support from teachers	.04	−.10*	.05	.09*	.38**							
T1 Support from best friend	−.18**	−.12**	.08	.17*	.11**	.08						
T1 Sexual harassment	−.17**	.02	−.04	−.11*	−.18**	−.17**	.02					
T1 Emotional problems	−.38**	.02	−.14**	−.13**	−.31**	−.29**	−.01	.29**				
T1 Well-being	.20**	−.04	.14**	.25**	.32**	.29**	.12**	−.23**	−.57**			
T2 Sexual harassment	−.17**	−.02	.01	−.09*	−.07	−.13**	.03	.35**	.18**	−.18**		
T2 Emotional problems	−.40**	.04	−.14**	−.08	−.26**	−.23**	−.04	.25**	.67**	−.47**	.19**	
T2 Well-being	.20**	−.03	.15**	.14**	.25**	.30**	.01	−.22**	−.48**	.58**	−.15**	−.54**

Note:* p < .05 ** *p* < .001.

### Cross-lagged model

**Figure 2.**
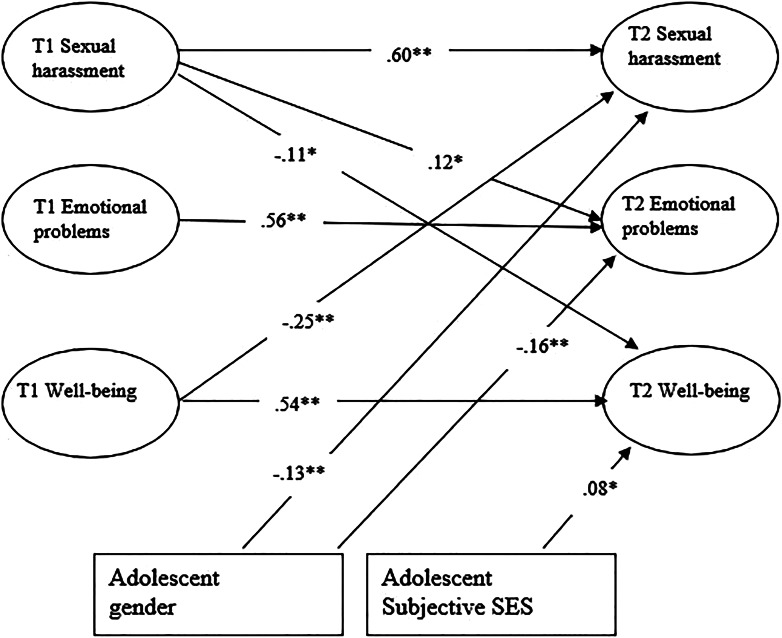
Overview of significant cross-lagged effects among latent factors of sexual harassment victimization, emotional problems and well-being, controlling for adolescent gender and subjective socioeconomic status (SES).

As shown in Figure 2, sexual harassment, emotional problems, and well-being were relatively stable over time. In terms of cross-lagged effects, T1 sexual harassment was positively related to higher levels of T2 emotional problems (*β* = .12, *p* = .020) and lower levels of T2 well-being (*β* = .11, *p* = .049). Moreover, T1 well-being was related to lower levels of T2 sexual harassment (*β* = −.24, *p* = .044), indicating a bidirectional link between sexual harassment and well-being. In terms of controlling the effects of adolescent gender, family structure, and SES, only adolescent gender and SES emerged as significant predictors. Adolescent gender was negatively related to both T2 sexual harassment (*β* = −.13, *p* = .016) and T2 emotional problems (*β* = −.15, *p* < .001), indicating that girls experienced higher levels of T2 sexual harassment and emotional problems than boys. Higher levels of SES were related to higher levels of T2 well-being (*β* = .08, *p* = .020). No other paths were significant in the model.

### Moderation by relational support

To test whether the links between the three constructs—sexual harassment victimization, emotional problems, and well-being—differed between groups of adolescents reporting different levels of relational support, we applied four separate multi-group analyses to the final cross-lagged model. Support from classmates (Δχ^2^ (1) = 7.596, *p* = .006) moderated the link between T1 sexual harassment and T2 emotional problems. T1 sexual harassment predicted higher levels of T2 emotional problems for students with poor support from classmates at baseline (*β* = .26, *p* < .001), but the link was non-significant for students with high support from classmates at baseline (*β* = −.05, *p* = .590). Support from the mother, support from a best friend, and support from teachers did not moderate any of the links in the cross-lagged model.

## Discussion

The first aim of this study was to examine the longitudinal and bidirectional links between sexual harassment victimization and two aspects psychological health, specifically emotional problems and subjective well-being (i.e., life satisfaction and purpose and meaning in life), in early adolescence. In line with several prior studies (Dahlqvist et al., 2016; Hill & Kearl, 2011; Kaltiala-Heino et al., 2016), but not all (Vega-Gea et al., 2016), we found that girls were more exposed to sexual harassment than boys at both points in time. In the cross-lagged analyses, we controlled for the effect of gender, so that the findings would apply to both genders. The second aim of the current study was to test the proposition that relational support alters individuals’ capacity to adapt to and cope with challenges and stressors (Cohen & Wills, 1985; Masten & Barnes, 2018). Specifically, the current longitudinal study was designed to answer the hitherto remaining question of whether relational support moderates links between sexual harassment victimization and poor psychological health over time.

In line with growing research (e.g., Chiodo et al., 2009; Dahlqvist et al., 2016; Rinehart et al., 2017; Skoog et al., 2016), our cross-lagged model clearly revealed that sexual harassment victimization was stable and predicted higher emotional problems and lower well-being over time. Previous cross-sectional research indicates that sexually harassed adolescents develop depressive symptoms because they feel ashamed about themselves and have negative attitudes about their bodies due to the harassment (Li & Craig, 2019; Sagrestano, Ormerod, & DeBlaere, 2019). Being the recipient of unwanted sexual comments, grabbing, or touching is clearly distressing and persistently psychologically harmful, perhaps even more so for adolescents than adults because the former are at a vulnerable psychosocial developmental stage (Thapar, Collishaw, Pine, & Thapar, 2012). Moreover, the findings suggest that sexual harassment not only triggers emotional problems, but also reduces adolescents’ life satisfaction and purpose and meaning in life.

Concerning reciprocal links between sexual harassment victimization and poor psychological health, a couple of prior studies have found that adolescents displaying depressive symptoms and who self-harm are more likely to be sexually harassed over time (Dahlqvist et al., 2016; Marshall et al., 2013). It has been speculated that depressive symptoms may elicit negative reactions from adolescents’ peers because such symptoms signal vulnerability (Schacter & Juvonen, 2017). In contrast, the current cross-lagged analyses indicated a reciprocal link between sexual harassment victimization and well-being, but not emotional problems. In other words, high life satisfaction and purpose and meaning in life were linked to a lower likelihood of being sexually harassed over time. To our knowledge, this is the first study that has identified such a link in an adolescent sample. Given that emotional problems and subjective well-being are distinct but related constructs—the former measuring the negative and the latter the positive aspects of psychological health (Keyes, 2007) —this finding is highly interesting. Despite its novelty, this finding is supported by a prior longitudinal study which found that a higher level of life satisfaction protects against peer victimization on social media among young people (Frison et al., 2016). In line with the speculations presented by Frison et al. (2016), it is possible that high well-being is protective because it signals strength and is linked to better coping mechanisms. This could have to do with self-assertiveness and the adolescent's self-concept (Card & Hodges, 2008). Our measures also tap into the concept of meaningfulness. Meaningfulness is a core concept in human life (Baumeister & Vohs, 2002). It is about feeling connected to something larger than oneself and is an integral part of leading a happy life. It concerns feeling that life makes sense and having an influence over the course of one's life. Moreover, it can make people better able to transform a negative series of events into a positive outcome. Given that sexual harassment was defined as victims’ perceptions of unwanted sexual attention, an alternative explanation could be that adolescents with higher well-being are more prone to perceive sexual attention from peers as less negative and unwanted or to forget negative and unwanted sexual attention. People with high subjective well-being, in contrast to those with low subjective well-being, are better capable of quickly rationalizing and discounting negative experiences, and—most importantly—do not dwell on such experiences (Lyubomirsky, Boehm, Kasri, & Zehm, 2011). In other words, adolescents who “feel better” might be less likely to report sexual harassment than others because they are cognitively less affected by it. Nevertheless, the findings suggest that low subjective well-being can be added to the previously identified predictors of sexual harassment victimization in adolescence, and that assumptions and conclusions drawn in cross-sectional research (e.g., Bucchianeri, Eisenberg, Wall, Piran, & Neumark-Sztainer, 2014) about the temporality of the link between sexual harassment and well-being might need to be revised.

Given that support from others may help individuals adapt to challenges and threats to their psychological health (Cohen & Wills, 1985; Masten & Barnes, 2018), the study went on to explore the role of relational support in the longitudinal, bidirectional links between sexual harassment, emotional problems, and well-being. We focused on support from people most central to adolescents’ relationship systems, namely their parents, teachers, and peers (best friends and classmates) in eighth grade (i.e., at T1). Firstly, and in line with prior studies (Doty et al., 2017; Espelage et al., 2019; Kaltiala-Heino et al., 2016), we found that more relational support at T1 was linked to less sexual harassment victimization at T1 and T2. The friendship protection hypothesis has been used previously as an explanation for the link between friendship support and bullying victimization (Kendrick et al., 2012). While adolescents with poor relationships with their social networks (e.g., parents and peers) tend to be vulnerable to harassment from peers (Doty et al., 2017; Espelage et al., 2019; Kaltiala-Heino et al., 2016), high relational support seems to promote resilience and mitigate the likelihood of being victimized by peers. It could be that relational support from family, teachers, and peers gives adolescents feelings of strength and of being protected by others (Sandler, Miller, Short, & Wolchik, 1989), thereby making them less easy targets for victimization.

Moreover, this study makes a fundamental contribution to the literature by demonstrating the moderating role played by relationships with classmates in the link from sexual harassment to emotional problems. We found evidence indicating that high-quality social relationships in the classroom decrease the harm attributable to sexual harassment victimization in terms of emotional problems among adolescents. This is in line with some previous findings that peer support moderates the link between peer victimization and poor psychological health (Holt & Espelage, 2007; Thompson & Leadbeater, 2013). It is also in line with the study conducted by Davidson and Demaray (2007), which identified classmate support as a moderator of the link between bullying and internal distress. We found evidence for specificity in the protective role of relational support, since other sources of relational support (i.e., from parents and teachers) were found not to protect victims of sexual harassment from developing higher emotional problems. This is in contrast with some findings in the general peer victimization literature which indicate that support from parents, best friends, and teachers are all protective (Duong & Bradshaw, 2014; Elgar et al., 2014; Sapouna & Wolke, 2013). Several of these prior studies had a cross-sectional design, which might partly explain the differences in the findings. The differences may also be explained by the fact that although bullying and sexual harassment share common features, including the tendency to be based on power (Gruber & Fineran, 2008), they are also unique phenomena that adolescents experience differently (Gruber & Fineran, 2016).

There is further support in the literature for our finding that support from classmates, but not other sources of relational support, moderates the link between sexual harassment and emotional problems. In early adolescence, there is a shift in social focus from parents to peers (Bornstein et al., 2013). Adolescents are highly attuned to, and affected by, their peer group—for better or worse (Somerville, 2013). Moreover, adolescents spend more time in school with their classmates than in any other social context. A positive classroom climate has been identified as predictive for changes in major developmental domains, and a positive classroom climate is particularly important for the psychosocial health of vulnerable groups (Eccles & Roeser, 2011). Furthermore, Harter (2012) argued and demonstrated that different sources of support are relevant in different domains. In the adolescent peer context, which is the context of the form of sexual harassment studied here, support from peers thus ought to be particularly important for psychological outcomes. In light of this, and given that sexual harassment typically takes place at school (Espelage et al., 2016; Young et al., 2009), it is reasonable to think that a good social climate in the classroom, including the feeling that classmates help each other out, has a buffering effect on victims of sexual harassment. Being sexually harassed is hurtful and causes feelings of shame (Lindberg, Grabe, & Hyde, 2007), but apparently might hurt less when adolescents feel that they belong to and are supported by their core peer group. The finding that relational support from mothers does not moderate the link between sexual harassment victimization and poor psychological health corresponds to some other findings in the bullying literature (Holt & Espelage, 2007; Rothon, Head, Klineberg, & Stansfeld, 2011). It has been speculated that adolescents become more secretive and are less willing to talk to and ask for help from parents (Finkenauer, Engels, & Meeus, 2002). This might particularly apply to sexual harassment as adolescents tend to avoid discussing sexuality with their parents (Rote & Smetana, 2015). It is possible that relational support from parents, and maybe also from teachers, plays a different role at earlier ages (Harter, 2012) and in different contexts and domains, such as family separation and school exams.

None of the forms of relational support moderated the link in the direction from lower subjective well-being to sexual harassment victimization. Interestingly, Frison et al. (2016) also reported that relational support moderated the link in the direction from victimization to poor psychological health, but not the opposite direction link. Future research is needed to explore other possible moderators. Factors that might be worth investigating that have been connected to psychological health and sexual harassment victimization are pubertal timing and sexual harassment perpetration (Dahlqvist et al., 2016; McMaster et al., 2002; Skoog et al., 2016). In summary, relational support from classmates at T1 moderated the effect of T1 sexual harassment victimization on T2 adolescent emotional problems, but not the effect of low subjective well-being at T1 on T2 sexual harassment.

### Limitations and strengths of this study

This study had limitations that need to be considered when interpreting the findings. Several of the limitations concern measurement. In general, there are no comprehensive, published tests of the psychometric properties of the measures used in this study. One specific limitation concerns the somewhat limited scope of the measure of sexual harassment. Our measure tapped into two forms of sexual harassment—verbal and physical—but not into a third, which has been used in some prior studies (e.g., Chiodo et al., 2009), namely visual harassment (e.g., displaying or sending pictures or images). Furthermore, no data were collected on sexual harassment on social media. Given that much sexual harassment takes place online (Hill & Kearl, 2011; Taylor, Liu, & Mumford, 2019) and not directly in school, it is possible that its effects are moderated by support from sources other than those involved in direct harassment. It should be noted, though, that sexual harassment in different contexts overlaps substantially (Taylor et al., 2019). According to Ybarra, Mitchell, and Espelage (2012) 42% of sexually harassed adolescents experienced harassment both online and in real-life environments. Furthermore, our measures of relational support were somewhat different from one another. For instance, whereas the measures of support from the mother and best friend were concerned with support from individuals, the measures of support from teachers and classmates focused on the overall social climate in a specific relationship system. Also, the data on support from the mother were collected 1.5 years before the data on support from the other social sources. However, as well-designed research has shown that relational support from mothers is highly stable during the early adolescent years (Cortés-García et al., 2019), there are good reasons to think that it was the same when the other data on support were collected. Thirdly, all the data used in this study were self-reported, which increases the risk of biased estimates due to common method variance. However, it is unlikely that common method variance poses a serious threat to the validity of the conclusions drawn (Malhotra, Kim, & Patil, 2006). Using self-reported data to measure relational support means that we tapped into perceived support rather than actually received support. Previous studies have found that perceived support has a stronger protective effect than actually received support (Szkody & McKinney, 2019), which is why it might be particularly important to tap into.

The study's strengths include a longitudinal design, a relatively large sample size, novel research questions, and the differentiation between support from best friends and classmates.

### Implications for future research and practice

Many questions remain about the developmental consequences of sexual harassment in adolescence in general and the roles played by the relationships in which adolescents are embedded. Firstly, more research is needed to understand why there seem to be different links between sexual harassment victimization and two different aspects of psychological health (emotional problems and well-being). The different findings might reflect the fact that negative and positive aspects of psychological health (ill-health versus health) are distinct constructs rather than two ends of the same continuum (Keyes, 2007). Secondly, given that sexual harassment starts at young ages, at a mean age of 13 years according to retrospective reports of adult women (Menssink, 2018), studies need to include younger cohorts than those used in the current study to fully understand the developmental trajectories of sexual harassment during the entire process of sexual maturation during adolescence. Such research is in progress (Skoog, Holmqvist Gattario, & Lunde, 2019). Thirdly, different subgroups of adolescents may be more at risk of being sexually harassed by peers than others. For example, lesbian, gay, bisexual, transgender, and questioning young people are subjected to high levels of peer victimization, including sexual harassment, which has negative impacts on their psychological health (Hatchel et al., 2018). More studies on the buffering mechanisms between sexual harassment and its consequences in vulnerable groups of adolescents are needed. Fourthly, there is a general need for longitudinal studies of the mechanisms that underlie the links between sexual harassment victimization and its various outcomes.

This study has important implications for practice. School counsellors and clinicians should be aware that sexual harassment victimization might be one underlying cause of the emotional problems and low subjective well-being of some young adolescents. Although currently not the case (Gådin & Stein, 2019), all schools should make forceful efforts to be free of sexual harassment. Both process and effect studies indicate that classroom-based social and emotional learning programs can promote good relationships among students and enhance the classroom climate (Brown, Jones, LaRusso, & Aber, 2010; Kimber, Skoog, & Sandell, 2013). To the extent that such programs are effective in developing positive relationships in the classroom, it is possible that they can be used in an attempt to buffer against sexual harassment and protect the victims of sexual harassment from developing emotional problems as a result of their harassment. This may be a step towards creating a healthier environment for adolescents in school.

## Conclusions

The current study adds to mounting scientific evidence of the harmful psychological consequences of being sexually harassed in adolescence (Chiodo et al., 2009; Dahlqvist et al., 2016; Petersen & Hyde, 2013; Skoog et al., 2016) in showing that being sexually harassed predicts both increased levels of emotional problems and reduced levels of subjective well-being. In addition, the temporality of the link between sexual harassment and well-being appears to be more complex than what might have previously been assumed. The link between sexual harassment and well-being was found to be reciprocal. Being satisfied with and having a purpose in one's life seems to protect against sexual harassment over time. Another main contribution of this study is that it provides novel insights into what, in a developmental–relational context, may protect adolescent victims of sexual harassment from developing emotional problems. Consistent with resilience theory (Masten & Barnes, 2018; Masten et al., 1990) and the buffering hypothesis (Cohen & Wills, 1985), victims of sexual harassment who experience support from classmates and a positive social climate in the school classroom seem to be more resilient in relation to developing emotional problems. Interestingly, relational support from parents, teachers, and friends did not show such a buffering effect. Although the findings need to be replicated in different samples, this new understanding of the role of the social climate in the classroom is an important step in the development of effective interventions targeting the negative consequences of adolescent sexual harassment.
